# Ecological Meanings: A Consensus Paper on Individual Differences and Contextual Influences in Embodied Language

**DOI:** 10.5334/joc.228

**Published:** 2023-10-10

**Authors:** Agustín Ibáñez, Katharina Kühne, Alex Miklashevsky, Elisa Monaco, Emiko Muraki, Mariagrazia Ranzini, Laura J. Speed, Cosimo Tuena

**Affiliations:** 1 Latin American Brain Health Institute (BrainLat), Universidad Adolfo Ibáñez, Santiago de Chile, Chile; 2 Cognitive Neuroscience Center (CNC), Universidad de San Andrés and CONICET, Buenos Aires, Argentina; 3 Global Brain Health Institute (GBHI), University of California San Francisco (UCSF), California, US; 4 Trinity College Dublin (TCD), Dublin, Ireland, IE; 5 Potsdam Embodied Cognition Group, Cognitive Sciences, University of Potsdam, Potsdam, DE; 6 Laboratory for Cognitive and Neurological Sciences, Department of Neuroscience and Movement Science, Faculty of Science and Medicine, University of Fribourg, CH; 7 Department of Psychology & Hotchkiss Brain Institute, University of Calgary, CA; 8 Department of General Psychology (DPG), University of Padova, Italy; 9 Centre for Language Studies, Radboud University, NL; 10 Applied Technology for Neuro-Psychology Lab, IRCCS Istituto Auxologico Italiano, Milan, IT

**Keywords:** Embodied language, embodied cognition, contextual variability, individual differences, imagery, mental simulation, frames of reference, bilingualism, aging

## Abstract

Embodied theories of cognition consider many aspects of language and other cognitive domains as the result of sensory and motor processes. In this view, the appraisal and the use of concepts are based on mechanisms of simulation grounded on prior sensorimotor experiences. Even though these theories continue receiving attention and support, increasing evidence indicates the need to consider the flexible nature of the simulation process, and to accordingly refine embodied accounts. In this consensus paper, we discuss two potential sources of variability in experimental studies on embodiment of language: individual differences and context. Specifically, we show how factors contributing to individual differences may explain inconsistent findings in embodied language phenomena. These factors include sensorimotor or cultural experiences, imagery, context-related factors, and cognitive strategies. We also analyze the different contextual modulations, from single words to sentences and narratives, as well as the top-down and bottom-up influences. Similarly, we review recent efforts to include cultural and language diversity, aging, neurodegenerative diseases, and brain disorders, as well as bilingual evidence into the embodiment framework. We address the importance of considering individual differences and context in clinical studies to drive translational research more efficiently, and we indicate recommendations on how to correctly address these issues in future research. Systematically investigating individual differences and context may contribute to understanding the dynamic nature of simulation in language processes, refining embodied theories of cognition, and ultimately filling the gap between cognition in artificial experimental settings and cognition in the wild (i.e., in everyday life).

## 1. Introduction

Embodied theories of cognition claim that sensory and motor experiences meaningfully contribute to higher-order cognitive processes, including language processing (Barsalou, 2008; Pecher & Zwaan, 2005; Wilson, 2002). Such theories allow considering these cognitive processes engaged with one another and with sensory and motor neural systems and to be more consistent with how cognitive processes operate in situated, real-life scenarios outside the laboratory. Specific to language processing, embodied theories propose that simulations of prior sensory and motor experiences contribute to semantic appraisal and processing of single words, sentences, and more extensive texts such as narratives, via partial re-enactments in sensory and motor brain regions (Barsalou, 1999). Several theories maintain a crucial role for simulations in language processing (e.g., perceptual symbol systems, Barsalou, 1999; action-based language theory, Glenberg & Gallese, 2012; action-perception theory, Pulvermüller, 2013; for a review see Meteyard, Cuadrado, Bahrami, & Vigliocco, 2012). At the same time, simulation is thought to occur in a flexible, context-dependent manner (Barsalou, Santos, Simmons, & Wilson, 2008; Barsalou, 2019; Kemmerer, 2019). However, most literature in this field aims to demonstrate embodied effects in language processing rather than investigate the flexibility and dynamic nature of such effects.

It seems clear that symbolic processing should be grounded to a certain extent in sensorimotor experiences. This idea motivated countless experimental studies, many of which indeed found embodied effects in language processing. However, many studies failed to find such effects or found only tiny ones. Moreover, the direction of these effects was often unpredictable (e.g., Estes & Barsalou, 2018; Morey et al., 2021; Shebani & Pulvermüller, 2018). With substantial evidence that sensory and motor simulations contribute to language processing, research questions are now shifting to how these embodied effects translate to situated, real-world language processing and comprehension (e.g., Barsalou, 2019; García & Ibáñez, 2016; Hasson, Egidi, Marelli, & Willems, 2018). As embodied cognition research begins to consider more complex interactions between body and brain processes (sensory, motor, and interoceptive experiences) and external contexts (physical and social environments), a natural prediction is that individual and contextual variability in embodied effects may be the rule, rather than the exception (Barsalou, 2020).

This consensus paper considers individual and contextual differences as two critical sources of variability in language processing and suggests taking these sources into account to further refine embodied theories. We examine existing evidence for the effect of individual variability and contextual modulation of language involving sensorimotor experiences, imagery ability, simulation strategies, healthy aging, neurodegenerative diseases, and cultural and cross-linguistic differences. In addition, we consider how contextual effects, such as grammar of word stimuli or task framing, modulate embodied language processing. Then we assess how a deeper understanding of these sources of variability can make the embodied cognition approach more robust, clarifying the functional role of sensorimotor simulation in language comprehension. Finally, we propose future research directions to consider inter-individual variability and context necessary to gain new insights into the role in language processing and (in)variability of embodied effects in general.

## 2. Group and individual differences in sensory and motor experiences: Knowing without experiencing?

Suppose mental simulation involves the re-enactment of sensorimotor information extracted from real-world experience. In that case, individual differences in sensory and motor experience should lead to differences in mental simulation and subsequent language comprehension. One way to address this question is to investigate individuals with sensory impairments from birth and see how atypical perceptual experience during development impacts language comprehension. For example, embodied cognition predicts that individuals with congenital blindness should have different representations for vision-related concepts than sighted individuals. Strikingly, blind children and adults appear to possess knowledge about visual properties comparable to sighted children and adults (for review, see Bedny & Saxe, 2012). For example, blind and sighted participants performed almost identically in ordering animals by size and height (Kim, Elli, & Bedny, 2019). It is in line with the modality-invariant hypothesis of conceptual development, which proposes that a significant proportion of conceptual knowledge is abstract and therefore not dependent on perceptual experience (Bedny & Saxe, 2012). However, more fine-grained differences in conceptual knowledge between blind and sighted participants have been observed on closer inspection. In a card sorting task, while blind individuals were able to sort animals by shape and texture in a broad taxonomic manner (i.e., separating aquatic animals from four-legged animals), within these broad taxonomic categories, apparent differences between blind and sighted participants emerge: only sighted participants separated pigs, boars, and sheep from other four-legged animals, and blind participants were unable to group animals by color (Kim et al., 2019).

While this rich literature is informative about *what* blind individuals know about visual properties, it tells little about *how* they process visually-related concepts during online language comprehension. Although blind individuals know what colors particular objects have, they do not use color knowledge when making similarity judgments about those objects (Connolly, Gleitman, & Thompson-Schill, 2007). It is then not the knowledge about vision-related properties that differs between sighted and blind individuals but the salience of this information and its functional use in cognitive processing. On the other hand, no difference was found in online language processing between blind and sighted individuals (Bottini, Morucci, D’Urso, Collignon, & Crepaldi, 2021). Against predictions from embodied language accounts, during a lexical decision task, both blind and sighted individuals responded faster to multimodal words (e.g., *spherical*) and unimodal vision-related words (e.g., *blue*) compared to abstract words (e.g., *logic*), demonstrating a typical concreteness effect. Visual experience might not be critical to the concreteness effect but rather modality-independent differences in other properties of abstract and concrete words.

Beyond differences in visual experience, recent research has begun to explore language in people with anosmia, a lack of sense of smell. Brain imaging research demonstrated no differences in levels of olfactory activation to odor words (e.g., *rose*) between anosmics and controls (Han et al., 2020; Joshi, Han, Faria, Larsson, & Hummel, 2020). However, participants with anosmia had higher activation in semantic regions of the brain, which suggests that comprehension of odor language may require more significant effort in such participants. Behavioral data also indicate little difference between anosmics and controls. In a case study, one anosmic participant differed from controls in rating words’ olfactory associations, but not in how they narrated odor-relevant scenes (e.g., describing a florist, Reilly, Finley, Kelly, Zuckerman, & Flurie, 2021). Anosmics also do not differ in their online comprehension of odor-related words. Speed, Iravani, Lundström, and Majid, (2022) found no difference between acquired anosmics and controls in response time or accuracy in a lexical decision task and a semantic similarity judgment task for odor- (e.g., *incense*) or taste-related (e.g., *chocolate*) words. Surprisingly, anosmic participants remembered more odor-related words than control participants did. Overall, the finding that losing the sense of smell does not lead to severe impairments in odor-related language is in line with the proposal that odor representations are not critical to language comprehension (Speed & Majid, 2018a, 2020).^1^

Besides addressing sensory impairments, it is incumbent on future research to explore the effect of enhanced sensory experience on mental simulation. For example, wine experts with extensive training in the sense of smell are better at naming wine odors (Croijmans & Majid, 2016), imagining wine odors (Croijmans, Speed, Arshamian, & Majid, 2020), and remembering wine odors (Croijmans, Arshamian, Speed, & Majid, 2021) as compared to novices with no wine training. It raises the question of whether such experts would show an enhanced mental simulation of wine odor and better comprehension of language describing wine odor.

Similarly, expertise in a unique motor repertoire shapes individual’s motor representations. For instance, when professional ballet dancers viewed videos of their practiced ballet actions, their brain motor regions became more active, including the premotor cortex, intraparietal sulcus, right superior parietal lobe, and left posterior superior temporal sulcus (Calvo-Merino, Glaser, Grèzes, Passingham, & Haggard, 2005; Beatriz Calvo-Merino, Grèzes, Glaser, Passingham, & Haggard, 2006). Similar findings were demonstrated for expert musicians (Candidi, Maria Sacheli, Mega, & Aglioti, 2014) or tango dancers (Amoruso, Pusil, García, & Ibanez, 2022; Amoruso et al., 2017, 2014) who also anticipate errors before they occur and their brain observation network decodes expertise. The embodied cognition theory predicts that differences in motor expertise should also result in different mental contents when comprehending action language. In line with this, responses to sentences describing sport-specific situations that matched a performed action or a presented picture were facilitated in athletes and fans, but not novices (Holt & Beilock, 2006; Ong, Lohse, Chua, Sinnett, & Hodges, 2014).

These principles apply not only to concrete but also to abstract concepts. A close relationship between an individual’s handedness and spatial mapping of valence exists: right-handers tend to associate positive concepts, e.g., goodness or honesty, with the right side stronger than left-handers (Casasanto, 2009; the body-specificity hypothesis, see Casasanto, 2011). This effect is explained by motor fluency: people associate more successful outcomes with the movements performed by their dominant hand. This motor fluency effect is also dynamic: wearing an inconvenient ski glove on the right hand for just 12 minutes changes space-valence associations, resulting in right-handed participants demonstrating spatial mapping of valence similar to that of left-handed participants (Casasanto & Chrysikou, 2011). Cultural differences might also be at play: for instance, reading direction (left-to-right vs. right-to-left) shapes spatial associations of time and number (Casasanto & Bottini, 2014; Pitt & Casasanto, 2020; Shaki & Fischer, 2018). Conceptual representations also correlate with individual psychological traits. For example, Kaup et al. (2021) found that cross-domain associations between positive vs. negative events and past vs. future tense predict the individual level of optimism/pessimism.

To conclude, unique sensorimotor experiences, either congenital or acquired, or the result of specific training, such as sports, sometimes lead to differences in conceptual representations and language processing. However, these differences may be difficult to detect: the flexibility of the conceptual system makes use of compensation strategies, as missing semantic information could be extracted from other sources, such as word co-occurrences (Bottini et al., 2020), intact perceptual channels (Cattaneo et al., 2008), or emotion (Speed & Majid, 2020). Notably, while differences in experiences are relatively easy to identify or manipulate, variability in cognitive processing of those experiences (such as mental simulation or imagery strategies) is more subtle and might be considered a mediator between past sensorimotor inputs and linguistic tasks at hand. Moreover, a full sensorimotor simulation is not always necessary: in some contexts, activating surface linguistic properties might be sufficient. Whether deep sensorimotor simulation is required or not likely depends on the situational context, such as task demands. In the following sections, we will discuss these topics in more detail.

## 3. Individual differences in imagery: From aphantasia to hyperphantasia and synaesthesia

Mental imagery research has a similar history to that of embodied language processing, with debate on whether mental imagery consists of amodal representational units or whether it includes simulations of past motor and perceptual experiences (Anderson, 1978; Kosslyn & Pomerantz, 1977; Pylyshyn, 1973). Current evidence supports at least a partial role for perceptual activation in visual imagery (Pearson & Kosslyn, 2015; Pearson, Naselaris, Holmes, & Kosslyn, 2015) and motor activation in motor imagery (Jeannerod, 2006). For instance, overlapping patterns of activity are observed in V1 during visual perception and visual imagery. An algorithm trained on activity during visual perception can decode activity patterns during visual imagery (Pearson & Kosslyn, 2015). More broadly, several overlapping ventral visual areas, parietal, and frontal regions have been identified that are active during both visual imagery and visual perception (Dijkstra, Bosch, & van Gerven, 2019). Whether motor imagery and action observation activate neural paths similar to those involved in action execution is unclear (e.g., Buccino et al., 2001). A recent systematic review of neuroimaging studies has specifically shown a frontoparietal network common to action observation, action execution, and motor imagery. This network includes premotor, inferior parietal, and somatosensory areas (Hardwick, Caspers, Eickhoff, & Swinnen, 2018). Despite the similar contributions of motor and perceptual experience to both imagery and embodiment, there is little investigation into the shared mechanisms that may contribute to mental imagery and mental simulation in language processing. Some embodied cognition theories have proposed that mental imagery and mental simulation processes are fundamentally different due to the conscious nature of mental imagery versus the unconscious nature of simulation (Barsalou, 1999; Zwaan & Pecher, 2012). However, there is no consensus in the imagery literature regarding whether imagery can occur involuntarily or unconsciously (Jeannerod, 2001; Pearson et al., 2015). Consistent with this view, Willems et al. (2009) compared neural activity during single-word action verb processing and motor imagery and found no overlap in the regions engaged during verb processing (premotor cortex) and imagery (premotor and primary motor cortex). Yet, this study did not consider individual differences in motor imagery (e.g., ability or preferred use). Other embodied cognition theories propose that simulations during language processing use the same mechanisms as simulations during imagery (specifically motor imagery; Cayol & Nazir, 2020). Meteyard et al. (2012) suggest that differences between mental imagery and simulation during language processing may be related to the depth of lexical-semantic processing (e.g., single-word vs. sentences and narratives). They propose that embodied language processing may be more similar to imagery when engaged during sentence processing or narrative comprehension. This proposal is consistent with evidence from Moreno et al. (2015), who observed EEG activity characteristic of motor imagery (mu frequency desynchronization, Pineda, 2005) during action-related sentence processing.

Implicit instructions to use imagination when reading a story and individual’s vividness of imagery predict the ability to become absorbed in a story (Mak, de Vries, & Willems, 2020). Yet, it is unclear to what extent this increased absorption is due to increased mental simulation. Research on individual differences in mental imagery and their relationship to embodied language processing provides inconsistent results. Two studies tested the relationship between sensorimotor simulation effects and individual differences in visual imagery (Hirschfeld, Feldker, & Zwitserlood, 2012; Zwaan & Pecher, 2012). In a sentence-picture verification task (SPVT, Stanfield & Zwaan, 2001), participants judge whether an image corresponds to the one described in a previously presented sentence. By using this task, Hirschfeld et al. (2012) found that self-reported vividness of visual imagery modulated the amplitude of ERPs related to semantic processing. However, Zwaan and Pecher (2012) found no evidence of a relationship between the vividness of visual imagery and embodied effects related to shape, color, and orientation. Pecher et al. (2009) also did not find a relationship between individual imagery vividness and modality switch effects in language processing. Similarly, Speed and Majid (2018a) found no association between clarity of auditory imagery (Willander & Baraldi, 2010) and mental simulation of sound for auditory-related nouns (e.g., *typhoon*). Similar findings have been observed in numerical cognition. Horizontal spatial associations of numerical concepts have been demonstrated, with smaller numbers associated with the left space and larger numbers associated with the right space. However, the use of mental imagery tasks, for example, when participants are asked to imagine a ruler in front of them during magnitude classification, has no impact on these associations (see Wood et al., 2008, for a meta-analysis).

Several studies have investigated individual differences in motor imagery and embodied effects in language processing. Pavan and Baggio (2013) examined the relationship between the vividness of movement imagery and motion aftereffects elicited from a verb phrase judgment task. Motion aftereffects are familiar to everyone who has traveled in a train: prolonged movement in one direction results in the perception that presented later stationary patterns are moving in the opposite direction. Pavan and Baggio (2013) induced the motion aftereffect in their participants and then presented them with phrases implying movement in the opposite direction. Pavan and Baggio expected faster responses in processing phrases aligned with participants’ current mental simulation (motion aftereffect). However, the authors did not observe the motion aftereffect in language processing and found no relationship to individual differences in the vividness of movement imagery.

In contrast, Cayol et al. (2020) measured individual differences in a motor imagery aptitude score, i.e., the time it took participants to assess how difficult it would be to pour water from a cylinder into another container based on participants’ previous motor experience. The motor aptitude score was calculated for each participant, with higher scores indicating better motor imagery aptitude. Motor imagery aptitude scores were positively related to word definition performance, but only for highly imageable words, suggesting that better motor imagery aptitude has a beneficial effect on recruiting sensorimotor information during language processing.

Muraki and Pexman (2021) used a composite measure of motor imagery ability based on several self-report questionnaires and motor imagery tasks (e.g., the Test of Ability in Movement Imagery, Madan & Singhal, 2013; the Florida Praxis Imagery Questionnaire, FPIQ, Ochipa et al., 1997; the Vividness of Movement Imagery Questionnaire 2, Roberts, Callow, Hardy, Markland, & Bringer, 2008). They tested whether motor imagery ability was related to body-object interaction effect in a lexical decision task and a syntactic classification task. Body-object interaction effect consists of faster processing for words whose referents can be physically interacted with (e.g., *bowl*) compared to words whose referents are not available for physical interaction (e.g., *cloud*; Siakaluk et al., 2008). In a sentence-picture verification task, Muraki and Pexman also examined the relationship between motor imagery ability and object shape congruency effects. They found no relationship between motor imagery and body-object interaction effects in the lexical decision and syntactic classification tasks and no object shape congruency effects in the sentence-picture verification task. However, in exploratory analyses, the authors identified a significant relationship between the body-object interaction effect in the syntactic classification task and praxis (imagery of hand and body positions during object use). Participants with more accurate hand position imagery had a stronger body-object interaction effect. These results suggest that broadly measured motor imagery ability is unrelated to sensorimotor simulations engaged during language processing. However, there may be a relationship between specific types of motor imagery and specific types of simulations.

Another way to investigate the relationship between imagery and simulation during language processing is to consider aphantasia. Individuals with aphantasia (around 0.7% of the population, Zeman et al., 2020) cannot experience visual imagery, although their visual perception is intact. This lack of visual imagery also affects the ability to remember the past and imagine the future (Milton et al., 2021). Suppose the ability to re-enact visual representations without the original stimulus (i.e., visual simulation) is a critical component of language comprehension. In that case, individuals with aphantasia should show impairment or slow down during language comprehension. Research into aphantasia is relatively new, and there is a wealth of research questions still to be investigated. Still, the initial evidence suggests that reading experience in aphantasia may differ from individuals without aphantasia. Wicken, Keogh, and Pearson (2021) found no change in physiological response to frightening stories compared to a baseline condition in aphantasics, while control participants exhibited a heightened physiological response. The authors suggest that story-evoked arousal may depend on using mental imagery to simulate the story’s content.

At the other end of what could be described as a continuum of visual imagery, or ‘vividness spectrum’ (Zeman et al., 2020), are individuals with hyperphantasia, whose visual imagery is experienced as vividly as actually seeing (around 2.6% of the population, Zeman et al., 2020). While this topic is under-researched, we anticipate that language comprehension in hyperphantasia would differ from individuals with typical visual imagery vividness. Enhanced vividness of visual imagery might enhance mental simulation during language processing and corresponding improvements in memory for language, as well as strengthened emotional association (Wicken et al., 2021; Zeman et al., 2020).

Another phenomenon involving unique internal sensory representations is synaesthesia. Synaesthesia is a rare neurological phenomenon in which individuals experience idiosyncratic, automatic, and vivid associations between the senses (Galton, 1880). One of the most common forms of synaesthesia is grapheme-color synaesthesia, where individuals experience color sensations when they perceive letters or numbers (Baron-Cohen & Harrison, 1997). These associations may be driven by cross-activation of the brain’s sensory regions, caused by a lack of synaptic pruning (Hubbard & Ramachandran, 2005) or by disinhibited feedback (Grossenbacher & Lovelace, 2001). Some models of synaesthesia suggest that synaesthetic associations are semantically mediated (Chiou & Rich, 2014; Meier, 2013), meaning that synaesthetic associations are not only experienced in response to perception of a stimulus (e.g., to a letter or number), but in response to any form of conceptual activation (see also Hubbard, Brang, & Ramachandran, 2011). For example, seeing a letter, hearing a corresponding phoneme, or merely thinking about a letter can lead to a synaesthetic color experience (Rich, Bradshaw, & Mattingley, 2005).

Following a semantic account of synaesthesia, it could be predicted that synaesthetic associations are also experienced during language comprehension. Suppose synaesthetic colors are experienced when listening to a specific musical instrument (Cytowic & Eagleman, 2011). In that case, the same synaesthetic colors should be activated during comprehension of language describing the sound of that musical instrument. Initial evidence suggests a relationship between synaesthesia and language (Russell, Stevenson, & Rich, 2015; Speed & Majid, 2018b). For example, the colors experienced by individuals with odor-color synaesthesia, who experience colors when they smell odors, were shown to be driven by the name given to the odor (Russell et al., 2015). Importantly, odor-color synaesthetes were better at naming odors than control participants without synaesthesia (Speed & Majid, 2018b). This suggests that color associations become integrated into the conceptual representation of the odor, subsequently facilitating naming. Similar evidence also arises from studies on number-color synaesthetes. For these synaesthetes, experiencing a specific color can activate the corresponding numerical magnitude, thus biasing performance in magnitude-related tasks (Cohen Kadosh & Henik, 2006; Niessen, Fink, Schweitzer, Kluender, & Weiss, 2015). This type of bi-directional synaesthetic experience further suggests that the stimulus and its corresponding synaesthetic perception belong to the same concept (Ranzini & Girelli, 2019). Future research should aim to investigate whether synaesthetic associations also occur during language processing and, if so, how this affects comprehension.

To summarize, the evidence for a quantitative relationship between imagery and mental simulation in language comprehension is mixed: while some studies found a connection between these two processes, others did not. Studies with populations from the extreme points of the imagery spectrum, namely with people with aphantasia or hyperphantasia, might be beneficial in clarifying the issue. One qualitative distinction between imagery and mental simulation in language comprehension might be the automatic, subconscious nature of the latter. Clarifying the relationship between the two abilities is vital for understanding the cognitive basis of language comprehension and the nature of sensorimotor simulation in embodied cognition.

## 4. From normal aging to neurodegenerative diseases and brain damage

There is clear evidence for adult sensorimotor simulation, but less is known about the effects of aging on embodied language processes. Since sensorimotor experiences progressively change with age, mental representations used during language comprehension may be affected by age-related neurocognitive differences across sensory, motor, and interoceptive domains. For example, word imageability increases with age, probably because older adults associate more real-life experiences with each concept (Simonsen et al., 2013). In another study, participants were faster to respond to an image exactly matching that described in a sentence than to an image merely representing the entity mentioned in the sentence (a sentence-picture verification task, Hoeben Mannaert et al., 2019; Zwaan et al., 2002), and this effect was stronger in older participants than in younger participants (Dijkstra et al., 2004). It suggests that older participants construct more robust visual simulations of a situation when processing language. In line with this, spatial associations of numerical concepts (small and left vs. large and right) become stronger with increasing age (see Wood et al., 2008, for a meta-analysis).

Early evidence suggests that action-related sentence simulation (action-sentence compatibility effect, ACE; Glenberg & Kaschak, 2002) is preserved in healthy older people and individuals that suffer from Alzheimer’s disease (AD; De Scalzi et al., 2015), possibly due to spared sensorimotor activity in AD and normal aging. Along these lines, Reifegerste et al. (2021) found differences in processing motor semantics across ages: while processing of non-motor words declines with age, processing of motor words remains at the same performance level. However, the so-called body-part-as-object error (i.e., pantomiming the visual appearance of an object instead of its use) appears more frequently in older than younger participants (Peigneux & van der Linden, 1999), thus implying less reliance on motor simulation with aging. It is an example of visual dominance: visual information outweighs the role of proprioceptive and motor-related information in older adults and receives a higher priority in the case of a cross-modal conflict (see for review Costello & Bloesch, 2017). Overall, there appears to be a developmental trajectory with increasing reliance on visual information and decreasing reliance on motor information in performing cognitive tasks.

Studying the effects of brain dysfunction on embodied language processes is crucial to understanding the causal role of the cortical motor areas in language. Several studies have shown that pathological changes in the critical motor areas, for instance, due to motor neuron disease, Parkinson’s disease (PD), and lesions impair action verb processing (e.g., Arévalo et al., 2007; Bak, O’Donovan, Xuereb, Boniface, & Hodges, 2001; Bocanegra et al., 2017; Boulenger et al., 2008; Cardona et al., 2014; Cardona et al., 2013; Damasio & Tranel, 1993; García et al., 2017, 2016; Ibáñez et al., 2013; Kargieman et al., 2014; Melloni et al., 2015; Neininger & Pulvermüller, 2001). In particular, there is increasing evidence that the frontostriatal system is involved in representing action language (for a review, see Birba et al., 2017). In an influential classical experiment, Boulenger et al. (2008) studied PD patients off and on dopaminergic treatment. They used a lexical decision task with a masked priming paradigm. In the off condition, namely when the motor regions suffered from dopamine depletion, the patients had deficits in processing action verbs but not nouns. Moreover, levodopa intake improved the processing of action verbs but not concrete nouns. The results demonstrated that the motor system is functionally involved in processing lexico-semantic information in action verbs (Cardona et al., 2014; Fernandino et al., 2013). In a similar vein, Rodríguez-Ferreiro et al. (2009) found that PD patients had an impairment in their capacity to name actions compared to objects, showing that the impairment occurs in both language comprehension and production. Individual differences within patient groups have also been observed: Desai, Herter, Riccardi, Rorden, and Fridriksson, (2015) found that impairment in action language comprehension was correlated with individual reaching performance in stroke patients.

Patient studies are also informative in revealing the fine-grained details of mental simulation. PD patients, for example, have difficulty with action verbs related to fast actions, but not slow actions, indicating that speed is a component simulated during action language comprehension (Speed, van Dam, Hirath, Vigliocco, & Desai, 2017). Interestingly, PD affects the perspective from which the patient simulates an action. Humphries and colleagues (2016) showed that healthy older people represented most of the action gestures from an egocentric point of view. Conversely, PD patients simulated actions from an allocentric (third-person) point of view. The authors suggest that this could be a compensatory visuospatial mechanism for the impairment in the egocentric motor representation. Consequently, impairments in spatial cognition could affect motor and spatial simulation of sentences.

Further evidence for a causal contribution of sensorimotor areas to action-related language is provided by studies investigating the effects of a brain lesion on the processing of action-related language. In a recent study, Dreyer et al. observed deficits in processing tool nouns after lesions in motor-related dorsal pre- and postcentral gray and white matter (Dreyer et al., 2020). This finding is crucial because it shows effects of motor regions can be generalized over action-relatedness of different parts of speech (i.e., verbs, nouns) and rules out the possibility that motor areas are involved in processing verbs specifically, irrespective of their meaning (Dreyer et al., 2020).

Assessing embodied language and contextual modulation seems to provide selective markers of brain diseases impacting primary or secondary motor process (Abrevaya et al., 2016; Birba et al., 2017; Bocanegra et al., 2017; Cardona et al., 2014; Cardona et al., 2013; García et al., 2017, 2016; Ibáñez et al., 2013; Kargieman et al., 2014; Melloni et al., 2015). The use of naturalistic, contextual and ecological approaches to embodied language (Birba, Beltrán, et al., 2020; Birba, Vitale, et al., 2020; García et al., 2020; García & Ibáñez, 2014; Kogan et al., 2020; Trevisan et al., 2017) seem to be a powerful tool to identify selective deficits in neurodegeneration and other brain diseases (Baez et al., 2020; Calvo et al., 2019; Díaz-Rivera et al., 2022; Eyigoz et al., 2020; García et al., 2018; Moguilner et al., 2021; Suárez-García et al., 2021). Interoceptive deficits and their relation with contextual modulation is also an innovative embodied approach across brain diseases (Abrevaya et al., 2020; Birba et al., 2022; de la Fuente et al., 2019; Fittipaldi et al., 2020; García-Cordero et al., 2016; Gonzalez Campo et al., 2020; Salamone et al., 2021; Yoris et al., 2020). Studying clinical groups can also reveal how embodied theories of language processing can help clinical treatment. In clinical practice, treatment following brain damage demonstrates the strong link between motor actions and language (e.g., Everard et al., 2020).

Recent studies have focused on motor and language rehabilitation for people with aphasia (PWA) or on using motor and language therapy combined to treat neurological motor diseases. The motor system responds rapidly to newly learned action words, and other modality-specific areas respond to novel tool names learned through manipulation or observation (Bechtold et al., 2019; Bechtold, Ghio, Lange, & Bellebaum, 2018; Fargier et al., 2012, 2014; James & Swain, 2011; Kiefer, Sim, Liebich, Hauk, & Tanaka, 2007; Liuzzi et al., 2010). Rapid exposure to an unusual motor experience (while still part of the human motor repertoire) via motor or observational learning is enough to elicit a facilitation effect in an action verb judgment task (Beauprez, Blandin, Almecija, & Bidet-Ildei, 2020). Manipulation of motor experience in terms of action observation is reflected in the works by Marangolo and colleagues, showing that action execution or action observation can mediate the recovery of language in PWA (e.g., Marangolo et al., 2010; Marangolo, Cipollari, Fiori, Razzano, & Caltagirone, 2012). Not only does this result prove the flexibility of embodiment effects, but it also unveils the translational potential of embodiment theories. Lexical access in PWA without semantic deficits was improved after training with observed movements that correspond to familiar actions part of the human repertoire (e.g., ‘walking’ versus ‘raining’) or embedded in an environmental context where the referents of the action and its goal were visible (e.g., ‘woman getting in the train’ in real life vs. ‘woman paying at the cash register’ pantomimed; Gili et al., 2017; Marangolo et al., 2012). These findings provide preliminary results on how factors impacting the contextual presence of an embodiment effect can be exploited in what they call ‘action observation therapy’, and encourage the testing of new action-based treatments to recover language disease (Picano, Quadrini, Pisano, & Marangolo, 2021). Besides action observation therapies, other treatments have already been proposed, based on the interaction between the motor and language systems, including Semantic Feature Analysis therapy (Boyle & Coelho, 1995), personalized observation, execution, and mental imagery therapy (Durand & Ansaldo, 2013; Durand, Berroir, & Ansaldo, 2018; Durand, Masson-Trottier, Sontheimer, & Ansaldo, 2021), gesture production therapies (Goldin-Meadow, Nusbaum, Kelly, & Wagner, 2001; Rose, Attard, Mok, Lanyon, & Foster, 2013) and language-action therapies (Difrancesco, Pulvermüller, & Mohr, 2012; Stahl et al., 2018). In a similar vein, neuromodulation studies have shown that stimulating the motor cortex (Branscheidt, Hoppe, Zwitserlood, & Liuzzi, 2018; Meinzer, Darkow, Lindenberg, & Flöel, 2016) and even cerebellar and spinal cord (see Pisano & Marangolo, 2020) facilitate verb retrieval in PWA. While these results warrant further investigation of the role of the concomitant language therapy and the contextual factors (e.g., task demands), they certainly show how the reciprocal influence of motor and language processing can support clinical treatment (Monaco, Jost, Gygax, & Annoni, 2019).

In conclusion, while little is known about how simulation is affected in normal aging, there is increasing evidence that neurodegenerative diseases affecting the motor system disrupt action language simulation, supporting a clear role for the action system in action-language comprehension. In addition, preliminary evidence suggests rehabilitation treatments of motor and language-related impairments can be more efficient when combining motor-cognitive aspects. It motivates the further refinement of embodied theories of language processing to be sufficiently applied in clinical practice.

## 5. Context effects and individual differences in spatial aspects of language comprehension: Allocentric vs. egocentric simulation strategies

Spatial aspects of sensorimotor simulation and language comprehension have received considerable research attention (Barsalou, 2008; Beveridge & Pickering, 2013; Zwaan & Radvansky, 1998). The spatial grounding hypothesis states that action simulations during language comprehension are grounded in the spatial context (Beveridge & Pickering, 2013). One aspect of the spatial context concerns the frame of reference. Spatial frames of reference can be divided into egocentric (body-to-objects relations) and allocentric (object-to-object relations), or in a body-dependent and body-independent representation of the space (Ladyka-Wojcik & Barense, 2021). These frames of reference are involved in language comprehension (Beveridge & Pickering, 2013).

The role of individual differences in spatial simulation of action-related sentences was researched by Vukovic and Williams (2015). They found that individuals with a bias towards the egocentric frame were significantly faster to verify egocentric pictures than allocentric pictures only for the ‘You’ sentences. The allocentric preference group did not differ for ‘I’ or ‘You’ sentences (i.e., egocentric vs. allocentric picture perspective). They concluded that only individuals with an egocentric preference simulate action-related sentences from an egocentric point of view (the presence of linguistic factors only hampers the embodiment in the allocentric group). In the second set of experiments, they provided a spatial context and two actors speaking (i.e., one saying to the other an action-related sentence – “*I open the bottle*”, “*You open the bottle*”) on the PC screen. Findings show that both egocentric-allocentric preference groups can embody the speaker’s point of view when spatial context is provided. Lastly, when the speaking actor was looking toward the participant, the egocentric and allocentric individuals kept their preferred spatial perspective: the egocentric group adopted a perspective of one of the speakers, and the allocentric group adopted a perspective of an external observer. The study highlights the crucial role of spatial frame preferences and the spatial context (see Beveridge & Pickering, 2013) in mental simulation during language processing. They also suggested that the actors’ role (i.e., agent or receiver of the action) in the sentence and scene could be determinant factors that could hamper embodiment.

Links between spatial cognition and mental simulation also come from a variety of other different fields of research, including affordances (action possibilities, Gibson, 1979) or empathy (Brunyé, Ditman, Giles, Holmes, & Taylor, 2016). Some studies showed that language-based information could activate affordances (Tucker & Ellis, 2004), and this information is not spatially constrained (i.e., near and far from the body; Ferri et al., 2011). We propose that affordance processing induced by words could also depend on spatial frames of reference and individual proclivity. Concerning empathy, Brunyé and colleagues (2016) showed that the ability to adopt egocentric and allocentric perspectives during reading is modulated by individual propensities to be empathically involved (but see Stietz, Jauk, Krach, & Kanske, 2019). When taken together, these findings suggest that a complex approach to the relation between language simulation and spatial cognition is required to understand language comprehension. Most studies agree that when single words or single actor sentences are presented, the simulation is based on the first-person perspective. However, it is less clear which perspective is adopted when two actors are mentioned (Beveridge & Pickering, 2013; Morey et al., 2021). Future studies could explore how spatial frame proclivity impacts simulating action sentences with two actors (e.g., agent vs. receiver; “*Andy delivered the pizza to you/You delivered the pizza to Andy*”). Moreover, individual preferences in this task could also show differential strategy-dependent embodiment processes. Another suggestion for future research is to explore the impact of frame preferences on verbally presented affordances.

Finally, the understanding of action language and how it relates to spatial cognition frames can also be improved by learning from patients with specific diseases. Evidence from neuropsychological studies on spatial cognition found that the frames of reference can be impaired differently depending on the underlying neurodegenerative disease. Consequently, deficits in spatial cognition could impact how language is simulated and understood in normal and pathological aging. Considering this, the contribution of one frame or the other to action simulation (e.g., see Humphries et al., 2016) could be studied in populations where action language is impaired, and one of the frames is more impaired than the other one. For instance, AD and its prodromal stages affect the allocentric frames of reference (Serino, Cipresso, Morganti, & Riva, 2014; Tuena et al., 2021). Conversely, PD, dementia with Lewy bodies, and vascular dementia impact egocentric processing (Humphries et al., 2016; Lowry, Puthusseryppady, Coughlan, Jeffs, & Hornberger, 2020; Nedelska et al., 2017; Thurm et al., 2016). In addition, as some neurodegenerative diseases, such as frontotemporal dementias, parkinsonisms, and AD, lead to differences in naming of manipulable and non-manipulable objects (e.g., Cotelli et al., 2006), it could be interesting to investigate linguistic affordances in these populations.

Thus, here we have seen how language is also intertwined with other cognitive processes such as spatial cognition (Beveridge & Pickering, 2013). We have shown that inter-individual variability or proclivity in spatial cognition could affect action language (Vukovic & Williams, 2015). Preferences in adopting a particular spatial frame of reference (egocentric or allocentric) affect the simulation of action-related sentences and possibly language-based affordances. Importantly, under the same experimental conditions, different participants may prefer different simulation perspectives when comprehending the same linguistic expression. Future research should focus on factors that explain these differing preferences, examine how stable such preferences are, and how they are related to resulting performance in different tasks.

## 6. Context effects: No two tasks are the same

In general, modern theories of embodied language agree that mental simulation is flexible, and its activation may differ depending on context (Lebois, Wilson-Mendenhall, & Barsalou, 2015; Zwaan, 2014), and is also especially impaired in multiple brain diseases (Baez, García, & Ibanez, 2017; Ibañez & Manes, 2012). One contextual factor that can affect activation of mental simulation is the depth of semantic processing. Louwerse and Jeuniaux (2008) manipulated depth of processing via two tasks: lexical decisions on word pairs (shallow processing) or semantic judgments on the relatedness of word pairs (deep processing). Word pairs were manipulated in terms of iconicity (whether words were presented in a congruent configuration, e.g., *attic* above *basement*, or incongruent configuration, e.g., *basement* above *attic*) and semanticity (high: words that frequently co-occur in texts; low: words that infrequently co-occur in texts). Both iconicity and semanticity affected responses in the semantic judgment task, but the shallow lexical decision task was only affected by semanticity. The authors concluded that embodied simulations are therefore not always necessary and may not occur during shallow tasks. It is in line with the broader proposal that an adequate level of comprehension can be achieved in shallow semantic tasks via linguistic associations rather than fully-fledged mental simulation (Barsalou, 1999; Louwerse, 2011).

Another factor that can affect the presence of mental simulation is grammar. Bergen and Wheeler (2010) tested for the ACE (Glenberg & Kaschak, 2002) while manipulating the grammatical aspect of sentences. Participants were presented with sentences describing a completed action (perfect form, e.g., *Chris patted the cat*) or an ongoing action (progressive form, e.g., *Chris is patting the cat*) and had to decide if the sentences made sense or not. For progressive sentences, the typical ACE effect was observed: responses were facilitated when the direction of response (button press towards or away from the body) was congruent with the direction of motion implied in the sentence. However, this effect was not observed for the perfect sentences when the actions were described as completed. Thus, motor activation occurs for actions described as ongoing, but not actions described as complete, and this modulation of activation is driven by sentence grammar.

Complementary evidence for the role of grammar arises from studies on the effect of action language on grip force. Already 100 ms after the presentation of hand-related verbs, participants’ grip force significantly increases (Frak et al., 2010). Grip force also increases when participants listen to sentences like “*Fiona lifts the dumbbells*” describing motor action. This grip force change significantly correlates with changes in brain activity at the individual level (Pérez-Gay Juárez et al., 2019). No such increase is observed for sentences implying no motor action like “*Edmonde loves the flower bush in her garden*”. However, this motor effect is modulated by sentence grammar: it is weaker when the action verb is negated (“*Fiona does not lift the dumbbells*”, Aravena et al., 2012) or when the agent’s intentional state is in focus (“*Fiona wants to lift the dumbbells*”, Aravena et al., 2014).

The experimental task modulates flexible recruitment of semantic information, with more relevant information receiving higher priority and being recruited earlier. This flexibility has been tested by varying task instructions between participants who were provided with the same word stimuli in semantic categorization tasks. Tousignant and Pexman (2012) found that changing how the task decision was framed modulated the body-object interaction effect. When participants were asked to decide if a word was an entity, the anticipated body-object interaction effect was observed. However, no effect was observed when participants were asked to determine whether a word described an action. In an fMRI study, different task instructions in a semantic categorization task (i.e., participants were asked to decide if the word was an animal or a concrete thing) led to differential recruitment of neural regions, despite the two conditions using the same word stimuli (Hargreaves, White, Pexman, Pittman, & Goodyear, 2012). In the ‘is it an animal?’ task condition, regions associated with knowledge of living things (e.g., left fusiform and inferior temporal gyrus, bilateral middle temporal regions) were active to a greater extent than the ‘is it a concrete thing?’ condition. In contrast, during the ‘is it a concrete thing?’ condition, the bilateral motor cortex was activated to a greater extent than in the ‘is it an animal?’ condition. These studies suggest a top-down modulation of sensorimotor simulation during language processing (van Dam, van Dijk, Bekkering, & Rueschemeyer, 2012).

A similar line of investigation examined whether the task decision influences the modality of sensorimotor simulation engaged during language processing. Words, especially nouns, are often associated with several modalities of sensory experience (Lynott, Connell, Brysbaert, Brand, & Carney, 2020; Miklashevsky, 2018; Speed & Brysbaert, 2021; Speed & Majid, 2017; Vergallito, Petilli, & Marelli, 2020; Zhong, Wan, Ahrens, & Huang, 2022), and thus their representations are multimodal. van Dam et al. (2012) tested whether context would modulate the activation of either visual (left fusiform gyrus; FFG) or motor (left intraparietal sulcus; IPS) regions by changing the instructions in a go/no-go semantic categorization task to emphasize either color (respond only to words with referents that are green) or action features (respond only to words with referents related to foot actions). They observed greater activation in the left IPS when the task instructions emphasized action features but no change in the left FFG when the task instructions emphasized color features, suggesting that context only modulated the recruitment of motor simulations. van Dam et al. (2012) propose action information may be more sensitive to context because action features are more variant (e.g., one object can be associated with different actions depending on the context). In contrast, color features are static (e.g., the color of an object usually does not change in response to its context).

Not only linguistic properties of experimental materials are relevant for embodied effects, but also a complex interaction between linguistic and motor requirements during an experiment. García and Ibáñez (2016) suggested a neurocognitive Hand-Action-Network Dynamic Language Embodiment (HANDLE) model for describing an interplay between action language processing and motor actions usually required in experimental studies. The authors point to three key factors: (1) linguistic demands (cf. word processing vs. sentence processing); (2) the complexity of required motor movement (cf. a simple button press vs. gesturing or object grasping); (3) the timing between action language input and the movement. Only by considering the combination of all three parameters can one explain the presence or absence of embodied effects in a specific study, the strength of such an effect, and its direction (facilitation or inhibition). Although García and Ibáñez only consider motor language in their model, this situated framework can clearly be adapted for other modalities and semantic domains.

To summarize, variability in embodied effects is associated with the task at hand and experimental context. For single words, co-occurrence information might be enough for performing a shallow task. Deeper semantic tasks or extended contexts (such as a sentence or a narrative) are associated with stronger embodied effects. Again, sentential structure, for example, a negation of action, modulates embodied effects and might even diminish them. Broadly understood, a given task includes not only the language processing itself but also the response, which, if it consists of motor action, can interact with language semantics. Further clarification is also needed to determine if context-dependent recruitment is modulated top-down (i.e., selectively attending to the most relevant modality amongst all sensorimotor simulations) or whether it is modulated bottom-up (i.e., selectively simulating in only the most relevant modality or type of sensorimotor information).

## 7. Crosslinguistic differences and effects of L1 vs. L2

Individual differences can also derive from a particular language or languages spoken by an individual. Both the linguistic and extralinguistic knowledge are in part modulated by the language one speaks, or culture one belongs to (e.g., Bylund & Athanasopoulos, 2014). In the following, we will go through some examples of cross-cultural and crosslinguistic differences and their implications for embodied effects in language processing.

Social and cultural history are embedded in words and their meanings, in the use of the body, in beliefs about the word and its functioning. While words like ‘bread’ or ‘coffee’ may refer to the same entity in Dutch and German, the same words can evoke different representations for people living in different places, such as the United States or France (Adams, 2016; Grosjean & Li, 2013). For example, the use of one vs. both hands is strictly connected to social status in some but not other cultures. Korean participants show a different congruence effect for unimanual vs. bilateral responses depending on whether the object transfer is to someone of higher social status or not (Dennison & Bergen, 2010).

Individual cultural and linguistic experiences also affect cognition (Casasanto, 2016; Quesque et al., 2020). Cultural differences are reflected in distance perception or in how we anticipate motor effort (e.g., Soliman, Gibson, & Glenberg, 2013). Cultural differences can also modulate categorization of novel objects. Beauprez et al. (2019) tested Japanese and French participants. The latter were culturally less exposed to robots in their everyday lives and interacted more often with instrument-like robots instead of robots as communicative agents. A semantic facilitation effect after observing a robot performing a congruent action was observed in Japanese but not in French participants. In another study, Ghandari and colleagues (2020) asked participants to imitate the action observed on a video and judge the sensibility of a literal or metaphorical sentence describing an action either congruent or incongruent with the video previously displayed. While the Italian group presented the expected concreteness effect and a bigger congruency effect for the concrete sentences, Iranians presented a reverse congruency effect (inhibition), especially for concrete sentences. Iranian and Italian participants may have integrated the gesture and language system differently because of their different historical and religious backgrounds. In the past, gestures were forbidden for Persians and, until recently, moving hands while talking was considered impolite (Ghandhari et al., 2020). On the contrary, Italians have richer gesture productions (Colletta et al., 2015), and simulating movements of the sentences may have been a more rapid and costless process. Similar cultural modulation of gesture directionality is observed in South American indigenous communities (Núñez & Cornejo, 2012). Thus, culture-specific factors are not always purely distinguishable from more linguistic ones, as the latter are often correlated or considered as a cultural product (Casasanto & Bottini, 2014; Majid, Jordan, & Dunn, 2015).

Grammatical and lexical categories present crosslinguistic variability in referential meaning for different semantic categories, such as colors, body parts, containers, and spatial relations (Majid et al., 2015). Different languages use diverse prepositions related to a support-type or a containment-type of spatial relationship to refer to the same perceptual reality. Another difference is in the extension of a word meaning, for example, the inclusion of the ‘hand’ when referring to ‘arm’, which seems more likely to happen for Dutch vs. Japanese speakers, while the latter tend to include the ‘arm’ when referring to the ‘hand’ and not vice versa (Majid et al., 2015; see also Brown, 2013). Such differences can even be present within the same language family (Majid et al., 2015). Even common action words such as ‘washing’ do not fully semantically align across languages (Thompson, Roberts, & Lupyan, 2018). Linguistic diversity translates into linguistic relativity effects leading to different performances. Bylund and Athanasopoulos (2014) showed that both automatic and post-perceptual categorization tasks are influenced by linguistic diversity, including grammatical categories describing temporal (Athanasopoulos & Bylund, 2013) and spatial properties (Talmy, 1978). These differences influence other cognitive processes, including categorization, stimuli salience, and meaning representation (Bylund & Athanasopoulos, 2014; Lupyan, Rakison, & McClelland, 2007; Pavlenko, 2003). However, despite the abundant literature on the subject (e.g., Athanasopoulos & Casaponsa, 2020; Bylund & Athanasopoulos, 2014; Casasanto, 2016; Lucy, 2016; Wolff & Holmes, 2011), crosslinguistic diversity is not yet discussed through the lens of embodiment theories.

Sensorimotor language-driven activations have been observed for different languages, such as English, French, Italian, German, Finnish, and Chinese (Pulvermüller, 2013; Wu et al., 2013) and Sign Language of the Netherlands (Ortega & Ostarek, 2021). As much as theories on linguistic relativity and on cross-linguistic differences involve conceptual meaning (Jarvis & Pavlenko, 2007), how meaning representation is differently represented and modulated depending on the language should be part of embodied research too. Sensorimotor simulation during word comprehension may differ depending on the structures of a specific language. Effects of linguistic relativity in terms of embodiment effects can be studied by coupling the analysis of motion events or gestures (Della Putta, 2018, pp. 36–37) and kinematic studies (Gianelli, Marzocchi, & Borghi, 2017). Gianelli et al. (2017) showed that the modulation of motor action induced by the use of pronouns is language-dependent.

However, the picture becomes even more complex when considering bilingualism. The influence of a language may differ according to the level of competence (Bylund & Athanasopoulos, 2014). For example, speakers who typically encode motion manner in their L1 by using action words that emphasize manner (e.g., ‘hop’, ‘run’, ‘sprint’) are more likely to pay attention to such properties. However, if they acquired a second language (L2) later which does not share the same way of encoding movement, the extent to which they modify this tendency depends on how long they have been immersed in the L2 context (Park, 2020; see also Jarvis, 2016). Following this evidence, the putative comparison between languages suggested above, on how they encode movement and the relative embodiment effects, would assume another connotation in within-subject designs. Comparing languages and their embodiment effects in between-subject designs allows one to study embodiment from different angles or in different forms. Differences in spatial properties encoded in the verbs (or other elements of the sentence) in language X and language Y would not likely be the same as the ones found in the same languages acquired and experienced differently in the same participant (e.g., Ahlberg, Bischoff, Kaup, Bryant, & Strozyk, 2017).

L1 and L2 are usually acquired in different ways, which may have implications for their degree of embodiment. The L1 is grounded in rich physical interactions with the environment and people occurring during development (e.g., Pulvermüller & Fadiga, 2010). At the same time, L2 is typically learned in a classroom setting, with fewer interactions with the surrounding environment, often via the mediation of L1 or rote memorization (‘parasitic representations’, Li & Jeong, 2020). Thus, a late acquired L2 should be less embodied. Previous research on emotion processing showed weaker *affective* embodiment for a late and low proficient L2 (e.g., Caldwell-Harris, 2015; Pavlenko, 2012), also regarding *sensorimotor* activation (Sheikh & Titone, 2016). Along these lines, some authors have hypothesized and tried to demonstrate a difference in the degree of embodiment of L1 versus a late acquired L2 (e.g., Foroni, 2015; Vukovic & Shtyrov, 2014). However, studies focusing on learning an L2 or an artificial language indicate that adults can have rapid, newly learned embodied simulations through sensorimotor experience (Kogan, Muñoz, Ibáñez, & García, 2020) or even through solely linguistic experience (Günther et al., 2020; see also grounding contagion in Pulvermüller, 2013). Still, this does not mean that embodied representations are activated in every context or that are similar between L1 and L2.

Other authors, according to the influential models of bilingualism (e.g., the Distributed Feature model, de Groot, 1992; the Bilingual Interactive Activation Plus model, T. Dijkstra & van Heuven, 2002; the Sense model, Finkbeiner, Forster, Nicol, & Nakamura, 2004; the Revised Hierarchical Model, Kroll & Stewart, 1994; Kroll, Hell, Tokowicz, & Green, 2010; the Modified Hierarchical Model, Pavlenko, 2009; or the more recent Multilink, T. Dijkstra et al., 2019), argued that L1 and L2 share or partly share the conceptual store and that this would entail a similar degree of embodiment. A similar degree of embodiment between L1 and L2 could also be expected if L2 is mediated by L1. There can be a transfer in meaning from L1 (Pavlenko, 2009) or the coactivation of L1 during lexical access (T. Dijkstra & van Heuven, 2002). At present, however, it is unclear how embodied language processing fits into existing models of bilingual semantic representation. This is mainly due to the disagreement on or lack of specification of the semantic categories in terms of format, components (e.g., features language-dependent and language-independent), and corresponding brain correlates (e.g., localist vs. distributed). Although the existing models partly support the above predictions, the brain mechanisms behind the complex relationships between L1 and L2 embodiment are not yet well understood as compared to monolinguals (see Pulvermüller, 2018).

While the mechanisms are unclear, evidence has started to accumulate in favor of an embodied L2. The few studies comparing the degree of embodiment in L1 and L2 have been reviewed from different perspectives (Kogan, Muñoz, et al., 2020; Kühne & Gianelli, 2019; Monaco et al., 2019) with the interim conclusion that L2 is embodied, but with contrasting evidence on the degree of similarity with L1 and several aspects needing further investigation. One of these is the timing of embodiment effects and relative language implication. Following this recommendation, Monaco et al. (2021) reported a difference in the motor language interaction between L1 and L2 at the early stages of word recognition. They suggested that those differences can vary according to which direction of the motor language processing interaction is investigated (motor–to-language/semantic resonance or language-to-motor/motor resonance), the stage of linguistic processing manipulated (early versus late), and the consequent activation of L1 or L2 network (e.g., early coactivation of L1 and L2 vs. late activation of L2). All these methodological choices are even more important considering that greater motor cortex activation was found in L2 compared to L1, independent of the motor relatedness of the stimuli (Monaco et al., 2021; Rüschemeyer, Zysset, & Friederici, 2006; Tian et al., 2020). Recent fMRI studies showed that motor implication within the structural connectivity of L1 and L2 differs as well: similar L1 and L2 induced motor activation have been observed, but the L2 network showed less integrated embodied connections (Zhang, Yang, Wang, & Li, 2020) or a different pattern of those (Tian et al., 2020).

In summary, embodiment effects have been found across languages and cultures, albeit with differences. A second language is grounded as well, possibly to a different extent compared to L1. Not all the studies have been able to show such differences though, and this is not surprising given the amount of modulating variables to consider. On top of the individual and contextual effects, the dynamicity of the language experience in a bilingual over time needs to be considered. Furthermore, differences between L1 and L2 seem to be persistently confounded by differences between languages. Cultural and language diversity, as well as bilingual evidence, should be better assessed within the framework of embodied theories. Methodological choices in terms of task, design, and population are also major determinants. Therefore, a change in perspective should take place: from aiming at questioning the generalizability of the embodiment effect to aiming at exploring its flexibility depending on the cultures, languages, and how their schema changes over time and over the experiences.

## 8. General Discussion

In this work, we reviewed multiple sources of evidence suggesting that the embodied cognition account requires refinement: specifically, the vast variability of embodied effects should be properly tackled. We outlined inter-individual differences and contextual influences as two main sources of variability, and how these can be taken into account to explain the outcomes of many embodied cognition studies (differences in sensorimotor experiences, imagery ability, use of various simulation strategies, and varying contextual influences and task requirements). Here below, we further discuss the importance of a deeper understanding of these sources of variability to better specify the embodied cognition theory and clarify the functional role of sensorimotor simulation in language comprehension. We also advance some recommendations for future studies on how to consider inter-individual variability and context, and we discuss the need to fill the gap between cognition in experimental settings and everyday life contexts.

In this review, we have seen how the magnitude and direction of embodied effects in language processing appear to be a function of individual expertise, biographical, and cultural factors, as well as contextual frames that can draw attention to a specific aspect of an object or situation. Exploring these nuances of embodiment remains a critical focus (e.g., Chatterjee, 2010; Hauk & Tschentscher, 2013; Kemmerer, 2015). But why is it important to study these individual and contextual variabilities? Semantic memory supports action, reaction, and adaptation to the environment (Barsalou, 2020), which requires a flexible semantic network. As put by Connell and Lynott (Connell, 2019; Connell & Lynott, 2014), concepts are not static, but dynamic processes which take into account attentional, sensorimotor, linguistic, situational, and affective characteristics of the moment. Since concepts need to be flexible enough to “*operate continuously in a noisy environment with limited resources*” (Connell, 2019, p. 1310), their grounding is not evident to the same extent in every given instance. This dynamic processing is also influenced by an individual’s unique constellation of physical, social, and cultural experiences. To address these ‘contextual dependencies’ (Gianelli, 2018) and individual differences, embodiment research must account for these factors when examining general embodiment effects and further test how these factors are related to flexibility in embodied language processing.

Testing and analyzing the modulating factors of context and individual differences should be done systematically. To do so, embodiment researchers will need to address the challenge that Hedge and colleagues (2018) described as a methodological misalignment between experimental research and correlational studies of individual differences. While classical experimental tasks are considered reliable when individual differences are at a minimum, the individual differences research uses measures that maximize discriminability between participants. The authors call this phenomenon the reliability paradox. The reliability paradox does not mean that integrating experimental studies and research on individual differences is impossible. Instead, it might only mean that new experimental tasks should be developed to capture inter-individual variability reliably. This might be achieved in two ways: first, by collecting individual measures as covariates and increasing sample sizes accordingly, as individual differences exploration requires; second, by designing experimental tasks and developing statistical methods for data analysis that are sensitive to individual characteristics of participants. Indeed, examining individual differences is not only important for a deeper understanding of psychological phenomena but is also essential for estimating the generalizability of these phenomena. For instance, Cipora and colleagues (2019) demonstrate that the spatial-numerical associations of response codes (SNARC) effect, an association of smaller numbers with the left and larger numbers with the right side, which was replicated multiple times, is rather driven by a minority (< 45%) of participants. Yet, this number of participants is enough to find a reliable effect at the group level, leading researchers to general conclusions about human numerical cognition. Cipora et al. (2020) suggest that the link between spatial-numerical associations and individual mathematical abilities is modulated by other individual cognitive abilities, such as spatial skills, inhibitory control, or information processing efficiency. This observation leads us to consider that also well-established phenomena disclosed by embodied language studies might be representative of subcategories of participants depending on specific subsets of cognitive factors. Nevertheless, reliability at the individual level has not been systematically explored in the context of embodied effects in language processing.

At the same time, however, for some research questions, it might be necessary to reduce the influence of contextual and individual variability to clarify embodied effects. It may include controlling for more item- or condition-related variables influencing the motor-language interplay, such as the task, emotional and linguistic content, linguistic, motor, and attentional demands, and the timing between processes implicated in the task. It is essential to acknowledge that every aspect of a study is an experimental choice that can influence the processes under investigation. Consistent with this, some authors (e.g., Gianelli et al., 2017; Monaco et al., 2019) have pointed out that capturing nuanced differences in embodiment and language use requires careful consideration of the design and methodology choices. For instance, the use of kinematic analysis to compare embodied effects across different languages (Gianelli et al., 2017), incorporating spatial and temporal precision to understand how embodied effects vary across different stages of lexical and semantic access. Additional insights may be gained by exploring linguistic-motor crosstalk in all aspects, such as the motor-semantic resonance (Monaco et al., 2021).

A more thorough understanding of the effect of contextual and individual differences on embodied processes in isolation will also be necessary to understand the complex interaction between these two sources of variability that arise in ecological settings. Our brains continuously engage with situated multimodal experiences involving internal and external influences. In every single cognitive process, action or interaction, we coordinate multiple processes in a synergic, spontaneous and mainly unconscious way (Ibáñez, 2018, 2019; Ibáñez et al., 2017; Ibáñez & García, 2018; Ibanez & Schulte, 2020). Imagine a social interaction with your partner and focus on your cognitive process: your brain was using visual and auditory attention, sensorimotor processes, memory, imagery processes, facial and emotional recognition, changes in body states (interoception), theory of mind, as well as language comprehension and production. These processes – constantly intertwined and automatically interconnected - create specific restrictions that allow for anticipation and understanding of different meanings. Even if each of these processes can be phenomenologically (and experimentally) differentiated and connected with discrete brain activity in the neuroscientific laboratories, these are analytical abstractions of a synergic and holistic blending of interdependent processes. There is a huge difference between cognition in the wild (i.e., everyday behavior) and the domesticated and compartmentalized cognition of neuroscientific (and cognitive) laboratories. Thus, we need a truly embodied and situated approach connecting the gap between the cognition in the wild and the isolated, compartmentalized understanding of the embodied cognition upheld by traditional approaches. Considering the role of inter-individual differences and context in future studies can contribute to reduce this gap.

## 9. Conclusion

The systematic study of individual and contextual differences will contribute to understanding embodied language processing in multiple ways. First, characterizing individual differences will lead to better generalizability of research findings by identifying clusters of individuals that reliably demonstrate particular embodied effects. Second, these investigations will also better translate embodied language processing into the clinical domain and learning studies. Understanding the variability of embodied effects in language processing is crucial for unfolding the applied potential of the embodied theory. Which effects are functionally relevant, and which are not? In what contexts do they emerge? For which groups of participants are they relevant? These questions need to be answered to develop scientifically grounded practical implementations. The use of specified embodied approaches for target groups and settings will maximize economic effort in education and therapy. Finally, such investigations will allow for more refined predictions that will test the ability of embodied models to account for the flexible and dynamic nature of language processing in both laboratories and everyday life contexts.

## Footnotes

Note that rating studies demonstrate a cross-linguistic regularity: olfactory and gustatory modalities overall play the lowest role in language semantics (e.g., see for English: Lynott et al., 2020; Dutch: Speed & Brysbaert, 2021; Speed & Majid, 2017; Russian: Miklashevsky, 2018; Italian: Vergallito, Petilli, & Marelli, 2020; Chinese: Zhong, Wan, Ahrens, & Huang, 2022).
